# Influence of Occupational Exposures on Cytokine Profiles in Patients with Membranous Nephropathy

**DOI:** 10.1016/j.ekir.2026.107094

**Published:** 2026-09-25

**Authors:** Maxime Teisseyre, Mounir El Mai, Marion Cremoni, Barbara Seitz-Polski, IHMN Investigators, Maxime Teisseyre, Kévin Zorzi, Céline Fernandez, Marion Cremoni, Vesna Brglez, Noémie Jourde-Chiche, Bertrand Knebelmann, Thomas Crepin, Emilie Cornec-Le-Gall, Céline Lebas, Fitsum Guebre-Egziabher, Claire Rigothier, Thierry Krummel, Dominique Chauveau, Jean-Michel Halimi, Jean-Jacques Montseny, Guillaume Queuffeulou, Barbara Seitz-Polski

**Affiliations:** 1 Centre de Référence Maladies Rares Syndrome Néphrotique Idiopathique et Glomérulonéphrite Extra-Membraneuse, Centre Hospitalier Universitaire de Nice, Université Côte d’Azur, Nice, France; 2 Laboratoire d'Immunologie et de Thérapie Cellulaire, Centre Hospitalier Universitaire de Nice, Université Côte d’Azur, Nice, France; 3 Institute for Research on Cancer and Aging (IRCAN), UMR7284 CNRS INSERM U1081, Université Côte d’Azur, Nice, France

**Keywords:** cytokine, membranous nephropathy, nephrotic syndrome, occupational exposure

## Introduction

Membranous nephropathy (MN) is a rare autoimmune kidney disease mediated by autoantibodies directed against podocyte antigens, such as phospholipase A2 receptor type 1 (PLA2R1). MN exhibits marked clinical heterogeneity, with some patients developing thromboembolic complications or experiencing frequent relapses despite rituximab treatment, whereas others achieve sustained remission following B cell-targeted therapy.^1^

Several factors have been associated with a poorer response to rituximab, including high-grade proteinuria, and high anti-PLA2R1 antibody levels.S1^,^S2 In patients with severe nephrotic syndrome, urinary loss of rituximab may reduce drug exposure and contribute to incomplete or shorter-lasting B-cell depletion.S3^,^S4 In addition, the development of antirituximab antibodies has been associated with lower circulating rituximab levels, faster B-cell reconstitution, and an increased risk of treatment failure or relapse.S5^,^S6 Thus, the depth and duration of B-cell depletion may represent important determinants of therapeutic response in MN.S7 However, such heterogeneity may also reflect pathogenic mechanisms extending beyond humoral autoimmunity.

Although MN has long been conceptualized as a humoral autoimmune disease, accumulating evidence supports a dysregulation of cellular immunity. Patients with MN demonstrate reduced regulatory T cell (Treg) and T helper 1 (Th1) activity, alongside increased T helper 2 and T helper 17 (Th17) responses.^2^ Th17 cells secrete proinflammatory cytokines such as interleukin (IL)-17A, which promote immune cell recruitment and antibody production, thereby amplifying inflammation and contributing to the pathogenesis of autoimmune disorders.^3^

Environmental factors may promote immune dysregulation in genetically or immunologically predisposed individuals and thereby contribute to the development of MN.S8 Mercury is a well-established environmental trigger, particularly in the context of mercury-containing cosmetics; in a series of 41 patients with mercury-associated glomerular disease, MN was the most common histopathological pattern.S9–S11 Occupational contact with hydrocarbons and organic solvents has also been associated with MN and, among patients with established disease, with an increased risk of progression to kidney failure.S12^,^S13 Consistent with these observations, we previously reported that patients with MN were more likely to work in the construction sector and were more frequently exposed to occupational toxic substances, including asbestos, organic solvents, and lead than the general working population.^4^ Together, these findings support the hypothesis that environmental factors and occupational toxicants may contribute to MN development and modulate its immune phenotype.

In this study, we analyzed the cytokine profile of 112 patients with MN enrolled in a prospective multicenter cohort across 13 centers in France (NCT04326218). All patients had active disease and had received no immunosuppressive treatment within the previous 6 months. The cytokine profile was also assessed in a cohort of 320 participants from the general population (NCT04429594) in order to establish baseline reference values.

In patients with MN, occupations were categorized as service workers, industry workers, construction workers, and unemployed and/or retired individuals, as previously described.^4^ We hypothesized that occupational exposures, particularly in the construction sector, may promote innate immune activation and Th17 polarization, thereby contributing to cytokine imbalance in MN.

The detailed methodology is provided in the supplementary data (Supplementary Methods).

## Results

Cytokine profiles were assessed in stimulated peripheral blood mononuclear cells using a standardized assay to evaluate functional immune responsiveness (Figure 1 and Supplementary Table S1).^2^ Construction workers exhibited significantly higher IL-17A levels compared with all other occupational groups, with a median IL-17A level higher than that observed in the general population cohort (Supplementary Table S1). In addition, IL-6 and IL-1β, 2 key innate proinflammatory cytokines that promote Th17 differentiation, were significantly higher in construction workers compared with service workers.^5^ Further supporting innate immune dysregulation, IFN-α levels were also significantly higher in construction workers relative to both industrial workers and unemployed or retired participants. Regarding Th1-associated cytokines, IL-12p70 concentrations were significantly higher in construction workers than in both industrial workers and unemployed or retired participants. IFN-γ levels were elevated in construction workers compared with service workers and unemployed or retired participants. Within the T helper 2 pathway, IL-4 levels were significantly higher in construction workers than in industrial workers, whereas IL-5 concentrations did not differ significantly across occupational groups. Finally, IL-10, a regulatory cytokine primarily associated with Treg activity, showed no significant differences between groups.

**Figure 1:**
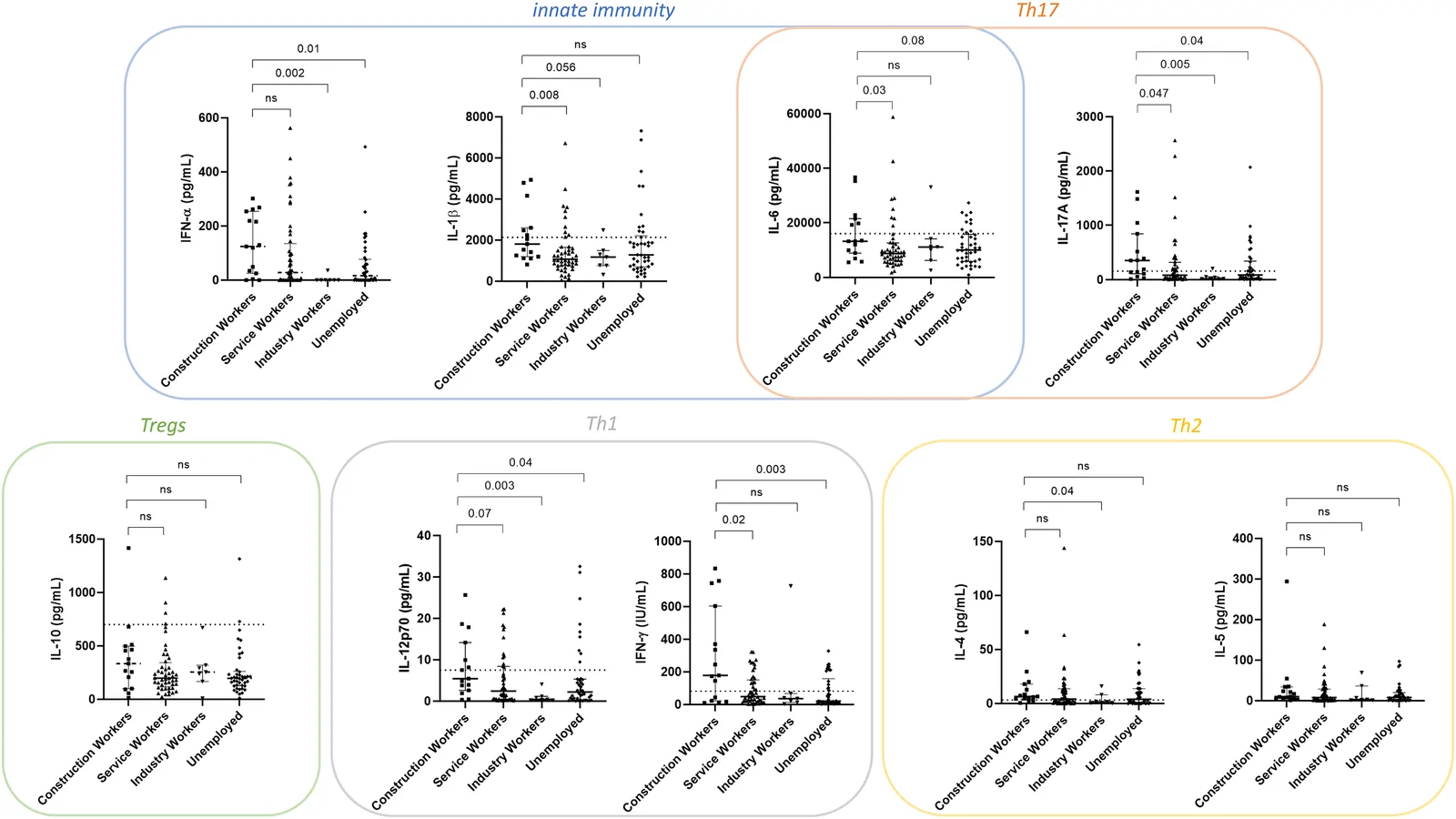
Cytokine profile according to occupational activity in patients with membranous nephropathy. Serum cytokine levels were analyzed in 112 patients with membranous nephropathy (MN) after *in vitro* stimulation of immune cells with anti-CD3 and a Toll-like receptor 7/8 agonist. The detailed methodology is provided in the Supplementary Data (Supplementary Methods). Occupations were categorized as service workers (*n* = 48), industrial workers (*n* = 7), construction workers (*n* = 15), and unemployed/retired individuals (*n* = 42), as previously described.^4^ Data are presented as median and interquartile range (Q1–Q3) and were compared with construction workers using the Wilcoxon–Mann–Whitney test. The dashed line indicates the median value derived from a cohort of 320 individuals from the general population. IFN, interferon; IL, interleukin; MN, membranous nephropathy.

To account for potential confounding factors, construction workers were matched 1:1 by age and sex to each of the other occupational groups. After matching, IL-17A levels remained significantly higher in the construction group compared with service workers (351.6 [109.1–844.3] vs. 79.1 [18.1–442.6], *P* = 0.03), industry workers (355.8 [27.6–1485.0] vs. 18.4 [13.3–51.2], *P* = 0.03), and unemployed/retired individuals (353.7 [136.8–762.3] vs. 28.8 [12.5–466.8], *P* = 0.044). In univariable analyses, construction-sector employment, sex, and serum albumin were associated with log-transformed IL-17A levels. In a primary multivariable model including construction-sector employment and sex (*n* = 112), construction-sector employment remained independently associated with higher IL-17A levels (*P* = 0.048). In a second model additionally adjusted for serum albumin (n = 106), the association was attenuated and showed a trend toward statistical significance (*P* = 0.055). In this fully adjusted model, construction-sector employment was associated with 2.5-fold higher IL-17A concentrations compared with nonconstruction employment (Supplementary Table S2).

The differences in cytokine profiles were observed despite similar markers of clinical and immunological disease severity across occupational groups. Urine protein-to-creatinine ratio, serum albumin levels, and anti-PLA2R1 titers were comparable between groups (Table 1). Estimated glomerular filtration rate was lower in the unemployed and/or retired group, likely reflecting their older age. Thus, the observed cytokine profiles appear to be associated with occupational exposure, independent of disease activity.

**Table 1:** Characteristics of patients according to occupational activity

Patient characteristics	Construction workers (*n* = 15)	Service workers (*n* = 48)	Industry workers (*n* = 7)	Unemployed/retired individuals (*n* = 42)	*P*-value
Age, yrs	56 (43–59)	58 (47–65)	37 (28–59)	72 (68–75)	< 0.0001
Gender, male/female	15/0	30/18	7/0	28/14	0.01
Weight (kg), median (Q1–Q3)	86.7 (74.5–93.4)	80.0 (68.0–93.0)	83.0 (80.0–96.0)	75.4 (67.0–88.0)	0.2
eGFR (ml/min/1.73m^2^), median (Q1–Q3)	72 (52–94)	75 (47–103)	73 (57–91)	38 (20–58)	< 0.0001
Albumin (g/L), median (Q1–Q3)	22.9 (20.1–33.0)	30.2 (23.3–35.6)	34.8 (19.6–39.6)	27.0 (16.7–31.0)	0.08
UPCR (g/g), median (Q1–Q3)	5.5 (2.3–7.1)	4.5 (2.2–6.9)	3.9 (1.1–5.2)	6.1 (4.3–8.1)	0.1
PLA2R1-associated MN, *n* (%)	12 (80%)	37 (77%)	5 (71%)	27 (64%)	0.5
Anti-PLA2R1 Ab (RU/ml), median (Q1–Q3)	45 (22–89)	49 (21–143)	46 (12–148)	84 (33–277)	0.2
Anti-PLA2R1 Ab epitope spreading Yes, *n* (%)	5 (42%)	17 (46%)	2 (40%)	11 (41%)	0.9

Ab, antibody; eGFR, estimated glomerular filtration rate; MN, membranous nephropathy; PLA2R1, phospholipase A2 receptor type 1; Q1, first quartile; Q3, third quartile; UPCR, urine protein-to-creatinine ratio.

## Discussion

Taken together, our findings identify a distinct proinflammatory cytokine profile in construction workers with MN, characterized particularly by higher IL-17A levels. The persistence of this difference after age- and sex-matching, together with comparable proteinuria, serum albumin levels, and anti-PLA2R1 antibody titers across occupational groups, suggests that this cytokine profile is not solely explained by differences in disease activity. This association is biologically plausible, as inhaled occupational toxic agents, including silica, asbestos, and organic solvents, can activate innate immune pathways through inflammasome signaling and induce type I and type II interferon responses, as well as T helper 2 and Th17 cytokine production.^6^, ^7^, ^8^, ^9^ These mechanisms provide a coherent framework for the cytokine signature observed in construction workers.

Our findings suggest that MN may not represent a uniform immunological entity, but rather a disease whose immune phenotype may be influenced by the environment. Although MN is primarily defined by an overactivation of humoral immunity, occupational exposures may influence upstream proinflammatory cellular immune cascades. Such immune amplification could contribute to clinical heterogeneity, including differential risks of relapse or thrombotic complications, previously associated with a Th17 proinflammatory cytokine profile.^2^

From a clinical perspective, the presence of cytokine differences despite comparable proteinuria, serum albumin, and anti-PLA2R1 antibody titers suggests that immune profiling may reveal heterogeneity not captured by conventional markers of MN activity. If validated, an occupational exposure-associated Th17-skewed profile could help identify patients at higher risk of adverse outcomes, support closer longitudinal monitoring, and complement conventional risk stratification. Longitudinal studies should determine whether this phenotype also predicts response to B-cell-depleting therapy.

This study has several limitations. Occupational category was used as a proxy for exposure; however, it does not capture cumulative dose, duration, or specific toxic agents and therefore may not accurately reflect exposure intensity. In addition, detailed information on previous occupations before retirement was not available for individuals classified as unemployed and/or retired, and previous occupational exposures may therefore have influenced their cytokine profiles. Although participants were matched by age and sex, residual confounding cannot be entirely excluded. The relatively small size of some occupational subgroups, together with the wide dispersion of cytokine values, may have limited the statistical power of some comparisons. No adjustment for multiple comparisons was performed, consistent with the exploratory and hypothesis-generating nature of the study. The observational design also precludes causal inference. Finally, the study was not designed to assess treatment response or longitudinal clinical outcomes. Nevertheless, the multicenter design enhances the robustness and external validity of the findings.

## Conclusion

In conclusion, occupational category may be associated with distinct cytokine profiles in MN, independently of disease activity. These findings support the concept that environmental exposures may modulate the immunologic phenotype of MN and highlight the potential importance of occupational health measures in patients with autoimmune kidney disease. Given the exploratory nature of this study, these findings require confirmation in a dedicated prospective study specifically designed to evaluate the impact of occupational exposure on cytokine profiles, ideally integrating quantitative exposure assessment and longitudinal outcomes to determine whether environmental immune amplification influences disease course.

## Appendix

### Members of the IHMN Investigators

Maxime Teisseyre, Kévin Zorzi, Céline Fernandez, Marion Cremoni, Vesna Brglez, Noémie Jourde-Chiche, Bertrand Knebelmann, Thomas Crepin, Emilie Cornec-Le-Gall, Céline Lebas, Fitsum Guebre-Egziabher, Claire Rigothier, Thierry Krummel, Dominique Chauveau, Jean-Michel Halimi, Jean-Jacques Montseny, Guillaume Queuffeulou, and Barbara Seitz-Polski.

## Disclosure

All the authors declared no competing interests in relation to this work.

## Supplementary Material

Supplementary file (PDF) **Supplementary Methods. Supplementary Reference. Table S1.** Description of cytokine profiles according to occupational activity in patients with membranous nephropathy and in the general population. **Table S2.** Multivariable analysis of factors associated with interleukin-17A levels.

## Footnotes

Supplementary file (PDF)
**Supplementary Methods**. **Supplementary Reference**. **Table S1.** Description of cytokine profiles according to occupational activity in patients with membranous nephropathy and in the general population. **Table S2.** Multivariable analysis of factors associated with interleukin-17A levels.
