# Supplementary material for: Influence of Occupational Exposures on Cytokine Profiles in Patients with Membranous Nephropathy
Source: Kidney Int Rep. 2026 Sep 25;11(12):107094. doi: 10.1016/j.ekir.2026.107094 (PMC13718639; doi:10.1016/j.ekir.2026.107094)
Supplement: Supplementary file 1 [file mmc1.pdf]

Supplementary Methods

Membranous nephropathy patient cohort

Patients with membranous nephropathy (MN) were enrolled between 2021 and 2025 in a prospective, multicenter cohort study conducted across 13 centers in France (NCT04326218). Inclusion criteria were: (i) biopsy-proven MN or confirmed by a positive serological assay in accordance with KDIGO and French guidelines (S14,S15); (ii) urine protein-to-creatinine ratio (UPCR) > 1 g/g; (iii) primary MN, defined by the absence of concomitant autoimmune disease, negative serology for human immunodeficiency virus, hepatitis B virus, and hepatitis C virus, and a negative malignancy workup; (iv) age ≥ 18 years; and (v) written informed consent.

Occupations were categorized as service workers (n = 48), industrial workers (n = 7), construction (n = 15), and unemployed/retired individuals (n = 42), as previously described (4).

General population cohort

A total of 320 participants from the general population cohort were enrolled between July 2020 and January 2021 (NCT04429594). The cohort comprised staff members from four sites: Nice Archet University Hospital, Nice Pasteur University Hospital, the Alpes-Maritimes Departmental Administrative Centre in Nice, and the municipality of La Trinité.

Ethics statement

All participants signed a written informed consent and the study adhered to the declaration of Helsinki.

Cytokine Analysis

For cytokine profile analysis, 1 mL of whole blood was stimulated with immune ligands in QuantiFERON™ tubes: anti-CD3 to activate T cells and R848, a Toll-like receptor (TLR) 7/8 agonist, to stimulate innate immune cells. With this assay, cytokine measurements represent induced immune responsiveness following stimulation, rather than basal circulating cytokine

levels. The samples were incubated at 37 °C for 16 hours, after which they were centrifuged at 3,000 × g for 15 minutes to separate the serum. In MN patients, cytokine concentrations were measured using a Meso Scale Discovery U-Plex® assay. In controls, cytokines were measured using custom-designed Ella cartridges (ProteinSimple™) or Meso Scale Discovery U-Plex® assay. To ensure comparability across platforms, cytokine concentrations obtained with the Ella system were converted to Meso Scale Discovery-equivalent values using previously established inter-platform correction factors. IFN-γ levels, were measured using Qiagen reagents on a DS2 automated enzyme-linked immunosorbent assay (ELISA) processing system.

Anti-PLA2R1 Antibodies

Anti-PLA2R1 titer and PLA2R1 epitope profile were determined with an ELISA from Euroimmun (Germany) and home-made ELISA, respectively.

Statistics

Categorical variables are presented as frequencies and percentages and were compared using Fisher's exact test or the chi-square test, as appropriate.

Continuous variables are presented as median and interquartile range. For comparisons between two groups, the Wilcoxon–Mann–Whitney test was used. For comparisons among more than two groups, one-way ANOVA was applied for normally distributed variables, and the Kruskal–Wallis test was used for non-normally distributed variables. Paired comparisons were performed using the Wilcoxon signed-rank test when individual matched pairs were available. When strict pairing could not be maintained, the Wilcoxon–Mann–Whitney test was used.

Because IL-17A levels were markedly right-skewed, a natural logarithmic transformation was applied before linear regression analysis. Factors associated with log-transformed IL-17A levels were first assessed using univariable linear regression. Variables considered clinically

relevant and associated with IL-17A levels in univariable analyses were subsequently evaluated in multivariable models. The primary model included construction-sector employment and sex (n = 112), while an extended model additionally included serum albumin (n = 106). Model assumptions were assessed using standard regression diagnostic plots.

All tests were two-tailed, and a P-value < 0.05 was considered statistically significant. Statistical analyses were performed with GraphPad Prism 8.4.3 (GraphPad Software Inc., San Diego, CA).

Table S1. Description of cytokine profiles according to occupational activity in patients with membranous nephropathy and in the general population

	construction workers (n = 15)	Service workers (n = 48)	Industry workers (n = 7)	Unemployed/ retired individuals (n = 42)	General population (n = 320)
IL-17A (pg/mL), median [Q1–Q3]	351.6 [109.1-844.3]	80.9 [19.7-318.6]	18.4 [13.3-51.2]	86.8 [16.5-341.3]	160.2 [25.5-317.6]
IL-6 (pg/mL), median [Q1–Q3]	13245 [8906-21505]	8777 [6222-12614]	11116 [6212-14138]	10003 [5877-15798]	16139 [10365-24189]
IL-1β (pg/mL), median [Q1–Q3]	1812 [1199-2606]	1072 [772-1658]	1179 [765-1496]	1285 [705-2209]	2141 [1425-3372]
IL-10 (pg/mL), median [Q1–Q3]	335 [100-498]	199 [117-343]	255 [167-320]	197 [125-262]	702 [476-951]
IL-12p70 (pg/mL), median [Q1–Q3]	5.4 [2.6-14.1]	2.4 [0.5-8.4]	0.4 [0.2-1.2]	2.2 [0.3-5.3]	7.6 [3.0-16.6]
IFN-γ (IU/mL), median [Q1–Q3]	179 [23-604]	49 [20-152] [a]	36 [13-68]	22 [9-159]	79 [15-197]
IFN-α (pg/mL), median [Q1–Q3]	125 [25-254]	28 [0-135]	0 [0-0]	17 [0-77]	NA
IL-4 (pg/mL), median [Q1–Q3]	6.4 [4.3-18.0]	4.2 [0.9-13.7]	1.7 [0.4-8.1]	3.9 [0.3-13.8]	3.2 [0.5-7.0]
IL-5 (pg/mL), median [Q1–Q3]	10.1 [5.2-34.3]	7.9 [2.0-28.7]	3.2 [1.3-36.4]	8.1 [1.4-20.2]	NA

Serum cytokine levels in 112 patients with membranous nephropathy (MN) and 320 participants from the general population after in vitro stimulation of immune cells with anti-CD3 and a TLR7/8 agonist.

Data are presented as median and interquartile range [Q1–Q3].

Abbreviations: IFN, interferon; IL, interleukin; NA, not available; Q1, first quartile; Q3, third quartile.

Table S2. Multivariable analysis of factors associated with interleukin-17A levels

	Univariable P-value	Model 1 β (95% CI)	Model 1 P-Value	Model 2 β (95% CI)	Model 2 P-Value
Age	0.35				
BMI	0.64				
Serum albumin	0.009			-0.03 (-0.07 to 0.002)	0.06
UPCR	0.96				
eGFR	0.83				
Anti-PLA2R1 Ab titer	0.35				
Sex (reference: male)	0.03	0.58 (-0.13 to 1.30)	0.11	0.43 (-0.32 to 1.18)	0.26
Construction-sector employment	0.02	0.95 (0.007 to 1.90)	0.048	0.91 (-0.02 to 1.85)	0.055

Because IL-17A levels were markedly right-skewed, a natural logarithmic transformation was applied before linear regression analysis.

Model 1: adjusted for sex and construction-sector employment.

Model 2: additionally adjusted for serum albumin.

Abbreviations: Ab, antibody; BMI, body mass index; eGFR, estimated glomerular filtration rate; IL, interleukin; PLA2R1, phospholipase A2 receptor; UPCR, urine protein-to-creatinine ratio.

Supplementary References

S1. Beck LH, Fervenza FC, Beck DM, Bonegio RGB, Malik FA, Erickson SB, et al. Rituximab-induced depletion of anti-PLA2R autoantibodies predicts response in membranous nephropathy. J Am Soc Nephrol. 2011 Aug;22(8):1543–50. doi:10.1681/ASN.2010111125 PubMed PMID: 21784898; PubMed Central PMCID: PMC3148709.

S2. Barbour SJ, Ronco P, Praga M, Fervenza FC, Induruwage D, Zhu B, et al. Predicting Remission in Antiphospholipase A2 Receptor Antibody-Associated Membranous Nephropathy: A Secondary Analysis of the GEMRITUX, MENTOR, and STARMEN Trials. Clinical Journal of the American Society of Nephrology. 2025 Jun;20(6):854. doi:10.2215/CJN.0000000694

S3. Teisseyre M, Cremoni M, Boyer-Suavet S, Crepin T, Benzaken S, Zorzi K, et al. Rituximab Immunomonitoring Predicts Remission in Membranous Nephropathy. Frontiers in Immunology. 2021;12.

S4. Allinovi M, Accinno M, Finocchi C, Mazzierli T, Caroti L, Lazzarini D, et al. Urinary rituximab loss and rate of treatment failure in membranous nephropathy. Nephrology Dialysis Transplantation. 2025 Jul 7;gfaf127. doi:10.1093/ndt/gfaf127

S5. Allinovi M, Teisseyre M, Accinno M, Finocchi C, Esnault VLM, Cremoni M, et al. Anti-Rituximab Antibodies Occurrence and Clinical Outcomes in Patients With Primary Membranous Nephropathy. Kidney International Reports. 2025 May 6;0(0). doi:10.1016/j.ekir.2025.04.059

S6. Teisseyre M, Allinovi M, Audard V, Cremoni M, Belvederi G, Karamé A, et al. Obinutuzumab and Ofatumumab are More Effective Than Rituximab in the Treatment of Membranous Nephropathy Patients With Anti-Rituximab Antibodies. Kidney International Reports. 2024 Dec 16;0(0). doi:10.1016/j.ekir.2024.12.012

S7. Teisseyre M, Cremoni M, Boyer-Suavet S, Ruetsch C, Graça D, Esnault VLM, et al. Advances in the Management of Primary Membranous Nephropathy and Rituximab-Refractory Membranous Nephropathy. Front Immunol. 2022;13:859419. doi:10.3389/fimmu.2022.859419 PubMed PMID: 35603210; PubMed Central PMCID: PMC9114510.

S8 Xu X, Nie S, Ding H, Hou FF. Environmental pollution and kidney diseases. Nat Rev Nephrol. 2018 May;14(5):313–24. doi:10.1038/nrneph.2018.11 PubMed PMID: 29479079.

S9. Li SJ, Zhang SH, Chen HP, Zeng CH, Zheng CX, Li LS, et al. Mercury-induced membranous nephropathy: clinical and pathological features. Clin J Am Soc Nephrol. 2010 Mar;5(3):439–44. doi:10.2215/CJN.07571009 PubMed PMID: 20089494; PubMed Central PMCID: PMC2827581.

S10. Narayanan R, Sivadas S, Kurien AA. NELL-1–associated membranous nephropathy linked to skin fairness cream use: insights from an Indian case series. Kidney International. 2024 Jun 1;105(6):1316–9. doi:10.1016/j.kint.2024.03.025

S11 Zhang L, Du M, Tu Y, Liang D, Wu X, Xie H. Clinicopathological features and long-term prognosis of glomerular diseases associated with mercury-containing cosmetics. J Nephrol. 2023 Jun;36(5):1401–7. doi:10.1007/s40620-023-01606-y PubMed PMID: 37060438.

S12. Gradden CW, Pai P, Hindell P, O’Donoghue DJ, Mason H, Bell GM. Membranous nephropathy, hydrocarbon exposure and genetic variants of hydrocarbon detoxification. QJM. 2001 Feb;94(2):79–87. doi:10.1093/qjmed/94.2.79 PubMed PMID: 11181983.

S13. Jacob S, Héry M, Protois JC, Rossert J, Stengel B. Effect of organic solvent exposure on chronic kidney disease progression: the GN-PROGRESS cohort study. J Am Soc Nephrol. 2007 Jan;18(1):274–81. doi:10.1681/ASN.2006060652 PubMed PMID: 17135394; PubMed Central PMCID: PMC4764668.

S14. Protocole National de Diagnostic et de Soins de la Glomérulonéphrite Extra-Membraneuse. 2022.

S15. Rovin BH, Adler SG, Barratt J, Bridoux F, Burdge KA, Chan TM, et al. Executive summary of the KDIGO 2021 Guideline for the Management of Glomerular Diseases. Kidney Int. 2021 Oct;100(4):753–79. doi:10.1016/j.kint.2021.05.015 PubMed PMID: 34556300.
